# The Positive Environmental Contribution of Jarosite by Retaining Lead in Acid Mine Drainage Areas

**DOI:** 10.3390/ijerph8051575

**Published:** 2011-05-13

**Authors:** Maria-Ondina Figueiredo, Teresa Pereira da Silva

**Affiliations:** Unity of Mineral Resources and Geophysics, Laboratório Nacional de Energia e Geologia (LNEG), Apartado 7586, 2721-866 Alfragide, Portugal; E-Mail: teresa.pena@lneg.pt

**Keywords:** jarosite, lead, environmental role, abandoned mine wastes

## Abstract

Jarosite, KFe_3_(SO_4_)_2_(OH)_6_, is a secondary iron sulphate often found in acid mine drainage (AMD) environments, particularly in mining wastes from polymetallic sulphide ore deposits. Despite the negative environmental connotation usually ascribed to secondary sulphate minerals due to the release of hazardous elements to aquifers and soils, jarosite acts as an efficient remover and immobilizer of such metals, particularly lead. The mineral chemistry of jarosite is reviewed and the results of a Fe *K*-edge XANES (X-Ray Absorption Near-Edge Structure) study of K-, Na- and Pb-jarosite are described and discussed within the context of the abandoned old mines of São Domingos and Aljustrel located in southern Portugal, in the Iberian Pyrite Belt (IPB).

## 1. Introduction

Secondary iron sulphates formed in abandoned sulphide-ore mines as a result of weathering in mine wastes—acid mine drainage (AMD) processes—currently have a negative environmental connotation because these secondary hydroxylated and/or hydrated minerals concentrate a large span of toxic elements, particularly the hazardous metals lead and thallium.

This apparently negative feature may become a positive contribution, since immobilization of such elements in the form of stable minerals significantly reduces their spread in soils and aquifers. This is just the case of the mineral jarosite—ideally KFe_3_(SO_4_)_2_(OH)_6_, a very common sulphate phase in AMD environments. The analysis of the chemical exergy of jarosite in relation to the uptake of lead has highlighted the possibility of a positive role played by this mineral in those environments [1] and subsequent X-ray absorption spectroscopy studies undertaken using synchrotron radiation [2,3] have provided clear evidence for the influence of the large cation upon the pre-edge details of Fe *K*-edge absorption spectra. The present work reappraises previous spectroscopic results and, within the context of waste materials from two abandoned old mines in the Iberian Pyrite Belt (IPB), reports a synopsis on the geochemical behaviour of jarosite susceptible of enhancing its environmental role.

### 1.1. An Overview on the Mineral Chemistry of Jarosites

Jarosite (*s.s*.) belongs to the alunite mineral group, having trigonal symmetry (space group R 3̄ *m* [4]) and general formula AB_3_(TO_4_)_2_(OH)_6_, where A represents a large cation with icosahedral coordination (coordination number CN = 12)—K^+^, Na^+^, NH_4_^+^, H_3_O^+^, Ag^+^, Tl^+^, Pb^2+^, Bi^3+^, plus minor Ca^2+^, Ba^2+^, Sr^2+^ and trivalent rare-earth ions, e.g., Eu^3+^, B stands for a smaller cation with octahedral coordination (CN = 6)—Fe^3+^ (jarosite *s.s.*) or Al^3+^ (alunite *s.s.*), plus minor V^3+^, Fe^2+^,Cu^2+^, Zn^2+^, Mg^2+^—and T represents a tetrahedral cation (CN = 4)—S^6+^, As^5+^, P^5+^. The octahedral B cations are surrounded by two oxygen anions from the tetrahedral groups (TO_4_) plus four hydroxyl ions (OH) shared between two neighbour octahedra; A cations are coordinated by six OH anions shared with the octahedral cations and six oxygens from the tetrahedral groups [5].

The alunite supergroup covers more than 40 distinct mineral species [6]. There is a limited solid-solution between end-members with respect to the octahedral B cations—jarosite (Fe) and alunite (Al)—and the same holds for the large icosahedral ions filling the A site—namely, K and Na, particularly for the ferric term.

In fact, the incorporation of lead within the crystal structure of jarosite is limited [7], despite the occurrence of a specific lead mineral—plumbojarosite, Pb_0.5_Fe_3_(SO_4_)_2_(OH)_6_—to which the same trigonal symmetry was attributed but with a double *c*-parameter of the hexagonal cell [8]. However, the need for lodging the lone electron pair of Pb^2+^ ions rises a space group ambiguity (centred R 3̄ *m* or non-centred R 3 *m* [1]), as quoted for the crystal structure of beudantite, Pb(Fe,Al)_3_[(As,S)O_4_]_2_(OH)_6_ [9] and further emphasized in a recent review on the nomenclature of the alunite supergroup [10].

The octahedral crystal field stabilizes the high-spin state ferric ions in the crystal structure of jarosite but the sharing of coordinating oxygen anions with large icosahedral cations is somehow expected to influence the electronic state of iron cations. This aspect can be suitably addressed by studying the X-ray absorption spectrum of iron at the *K*-edge, whose pre-edge details will reflect the potential variations in the electronic state of the metal ion.

### 1.2. A brief Account on Jarosite in Mining Wastes from IPB Polymetallic Sulphide Ore

The Portuguese sector of the Iberian Pyrite Belt (IPB) has been exploited since pre-Roman times and during the Roman occupation of Iberia, when gold and silver were extracted from extensive massive gossan caps, particularly the jarosite-rich layer at the base [11].

The modern exploitation of São Domingos mine in Alentejo (northern sector of IPB) started in 1859 for the extraction of copper along with these precious metals and since 1870 also for sulphur production until 1966, when the mine was definitely closed. Records on mine activity allow for estimating the extracted ore (mainly constituted by pyrite, chalcopyrite, sphalerite, galena, arsenopyrite and various sulphosalt minerals) at several million tons. The exploitation of the mining complex at Aljustrel started in 1847 and the mineral assemblage includes additionally tetrahedrite and tennantite. In both cases, it is nowadays envisaged to carry out a complete study of the potential sustainability of recovering scarce and valuable metals from these abandoned mine wastes, in line with EU recent efforts to promote new mineral resources through mining waste materials.

## 2. Experimental Section

Two lines of development were experimentally addressed: the susceptibility of iron electronic state to the population of the large icosahedral site and the effective retention of lead by jarosite in AMD processes.

### 2.1. Spectroscopic Study at the Iron K- Absorption Edge: Fe 1s XANES Spectra of Jarosites

X-ray absorption experiments at the iron *K*-edge were carried out at the European Synchrotron Radiation Facility (ESRF, in Grenoble, France) using the instrumental set-up of beam line ID-21 [12] by irradiating a small area (∼1 μm^2^) of well crystallized mineral fragments of K-, Na- and Pb-jarosites, previously studied by X-ray diffraction in the laboratory.

XANES spectra were collected in fluorescence yield (FY) mode with an energy-dispersive high-purity Ge-detector mounted perpendicular to the X-ray beam in the horizontal plane. A fixed-exit Si (220) crystal monochromator assuring an energy resolution of 0.3 eV at the Fe *K*-edge was used in the energy scans (7,050–7,350 eV). Metallic iron was scanned for energy calibration and pre-edge details of Fe *K*-edge XANES spectra were deconvoluted into pseudo-Voigt components with Fityk program [13].

### 2.2. Lead Retention by Jarosite in Waste Materials from Old Mines

Two type-case materials of waste products from old mining sites in southern Portugal were comparatively analysed with the aim of ascertaining the role of jarosite as an efficient Pb-retainer: a reddish dusty residue of roasted material collected at Aljustrel (sample 2–1) and a silty greyish material from a sampled profile at Achada do Gamo, São Domingos mine (sample 3–5).

Common X-ray laboratory techniques were applied to characterize these samples: X-ray diffraction (XRD) for phase identification and X-ray fluorescence spectrometry in wavelength dispersive mode (XRF-WDS) to assess the chemical constitution and comparatively estimate elemental contents.

A powder diffractometer with Bragg-Brentano geometry operating at 50 kV and 40 mA and equipped with a large-anode copper tube and a curved graphite crystal monochromator was used to collect X-ray diffraction patterns from sampled wastes.

A Philips PW1400 automated X-ray fluorescence spectrometer equipped with a rhodium tube and various analysing crystals was used to scan the angular intervals (2θ) suitable for element detection through the scanning of selected emission lines (Table 1).

Thermo-analytical techniques—simultaneous differential thermal analysis (DTA) and thermo-gravimetry (TG)—were applied to compare the thermal evolution of the samples using a SETARAM 92-16.18 apparatus that incorporates a microbalance with a controlled gas flow of argon (inert atmosphere). About 100 mg of sample were deposited in an alumina (α-Al_2_O_3_) crucible. The reference material was alumina powder and the temperature ranged from ambient to 650 °C at a heating rate of 10 °C min^−1^. After the DTA/TG test the phase constitution of the heated material was monitored by XRD and compared to the original mineralogical constitution in order to interpret the registered energy variations and mass losses.

## 3. Results and Discussion

**Figure 1.**
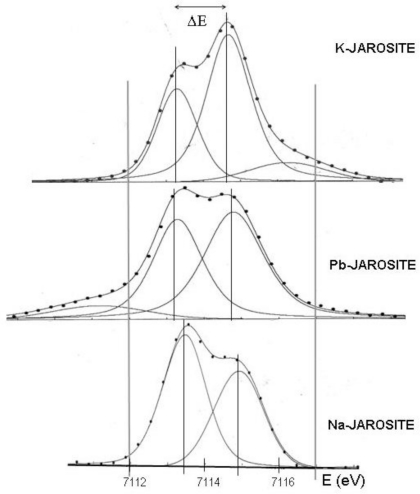
Pre-edge region of Fe *K*-edge XANES spectra collected from various jarosites.

The pre-edge features of Fe 1*s* XANES spectra from K-, Na- and Pb-jarosites are reproduced in Figure 1. As expected from octahedral ferric iron [14], the average pre-peak centroid is located at about 7,114 eV.

Conversely, the energies of the two pseudo-Voigt components (Figure 1) clearly depend on the size and speciation or electronic state of the large cation in icosahedral coordination by six oxygens from the sulphate tetrahedral groups plus six hydroxyl groups from iron octahedra.

A full justification of the observed differences falls beyond the scope of the present work but, in a general way, they may be explained considering that K^+^ and Na^+^ are closed shell, non-polarisable alkaline ions with different ionic radius—inducing a smaller *c/a* ratio for natrojarosite, while Pb^2+^ ions are off-centred within the icosahedron in order to accommodate the lone-pair of 6*s*^2^ electrons.

Concerning the chemical constitution of mining waste materials, the samples from São Domingos and Aljustrel display significant differences as ascertained by XRF-WDS (Table 2).

**Figure 2.**
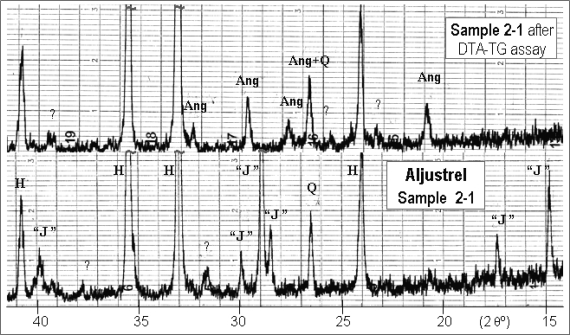
XRD patterns from Aljustrel sample, before and after the DTA-TG assay: Ang–anglesite (PbSO_4_, JCPDF card nr. 5-577); H–hematite (α-Fe_2_O_3_, 13-534); “J”–jarosite [KFe_3_(SO_4_)_2_(OH)_6_, 22-827]; Q–quartz (α-SiO_2_, 5-490); ?–weak lines (non identifiable).

The mineralogy of these materials clearly reflects the differences in elemental composition. The red mine waste from Aljustrel is composed of hematite and jarosite and does not comprise a specific lead-term (Figure 2).

Accordingly, this hazardous metal was expected to be incorporated in jarosite by partially replacing potassium, as confirmed by the presence of lead sulphate (anglesite) in the heated material resulting from the DTA/TG assay (Figure 2).

This behaviour matches the decomposition of a jarosite with low lead content (x much lower than 0.5, found in the mineral plumbojarosite) through water liberation and without S^6+^ reduction, according to Equation (1):

(1) $$2\;{\text{K}}_{1-2\text{x}}{\text{Pb}}_{\text{x}}\;{\text{Fe}}_{3}{\left({\text{SO}}_{4}\right)}_{2}{\left(\text{OH}\right)}_{6}\to 3\;{\text{Fe}}_{2}{\text{O}}_{3}\underset{︸}{+(2\text{x})\;{\text{PbSO}}_{4}+(1-2\text{x})\;{\text{K}}_{2}}\text{O}+6\;{\text{H}}_{2}\text{O}↑+(4-2\text{x})\;{\text{SO}}_{3}↑$$

**Figure 3.**
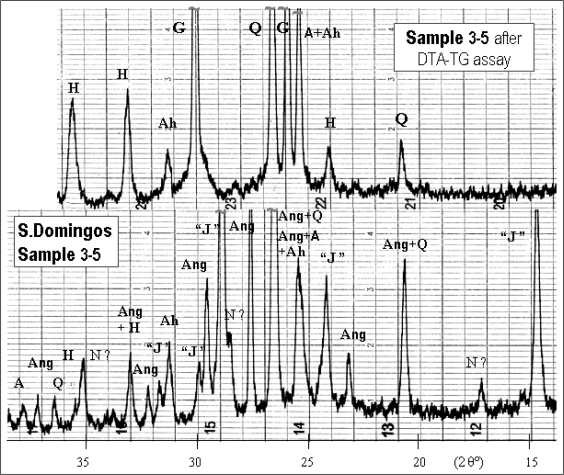
XRD spectra from São Domingos sample, as collected and after DTA-TG assay: Ang, H, “J”, and Q as quoted in Figure 2, plus A–anatase (TiO_2_, JCPDF card nr. 21-1272); Ah–anhydrite (CaSO_4_, 6-226); G–galena (PbS, 5-592); N?–natrojarosite, doubtful [NaFe_3_(SO_4_)_2_(OH)_6_, 11-302].

Conversely, São Domingos sample displays a more complex mineralogy that already includes anglesite, PbSO_4_. The phase constitution of DTA/TG heated material reflects the behaviour of this sulphate under a thermal treatment in inert atmosphere—reduction of S^6+^ to S^2−^ with PbS formation (Figure 3).

## 4. Conclusions

X-ray absorption spectroscopy has proven to be a most useful technique in the present minerochemical study. Indeed, Fe 1*s* XANES spectra collected from jarosite (*s.s*.), natrojarosite and Pb-rich jarosite provided a clear evidence about the influence of the large cation site population in natural minerals—a result that can be advantageously transferred to jarosites (*s.l*.) in mining waste materials.

Simultaneously, the thermoanalytical assays of illustrative examples of mining wastes from São Domingos and Aljustrel—whose phase composition and bulk chemical constitution were assessed by X-ray techniques—disclosed the reduction of natural lead sulphate when heated under an inert atmosphere.

As a whole, the above described results emphasize the retention of lead by jarosite in sulphide ore mine wastes, thus supporting data from previous authors, e.g., [15], about the efficient positive environmental contribution of this secondary iron sulphate in AMD processes.

## Figures and Tables

**Table 1.** X-ray emission lines adopted to characterize the elemental composition of mining waste samples.

**Element**	**K**	**Sn**	**Sb**	**Ca**	**Ba**	**Ti**	**Fe**	**Pb**	**Hg**	**Tl**	**Bi**	**Se**	**As**
Line	*K α*	*L α* 1	*L α* 1	*K α*	*L α* 1	*K α*	*K α*	*L* L	*L α* 1	*L α* 1	*L α* 1	*K α*	*K β* 1
2 θ° (Li F 200 crystal)	136.7	126.77	117.34	113.1	87.17	86.14	57.52	39.17	35.91	34.90	33.01	31.89	30.45
Collimator	Large				Fine								

**Table 2.** Comparative elemental contents of mining waste samples obtained by XRF-WDS (the number of crosses expresses the relative proportion; vtg, vestigious).

**Sample**	**K**	**Ca**	**Ti**	**Ba**	**As**	**Se**	**Sb**	**Sn**	**Bi**	**Tl**	**Hg**	**Fe**	**Pb**

**2-1** (Aljustrel)	x	x	x	x	x	vtg	vtg	vtg	vtg	vtg	vtg	xxxx	x
**3-5** (S. Domingos)	xx	xxx	xxx	vtg	xxx	x	x	xx	xx	vtg	vtg	x	xxxx
